# Schisandrin B Prevents Doxorubicin Induced Cardiac Dysfunction by Modulation of DNA Damage, Oxidative Stress and Inflammation through Inhibition of MAPK/p53 Signaling

**DOI:** 10.1371/journal.pone.0119214

**Published:** 2015-03-05

**Authors:** Rajarajan A. Thandavarayan, Vijayasree V. Giridharan, Somasundaram Arumugam, Kenji Suzuki, Kam Ming Ko, Prasanna Krishnamurthy, Kenichi Watanabe, Tetsuya Konishi

**Affiliations:** 1 Department of Clinical Pharmacology, Niigata University of Pharmacy and Applied Life Sciences (NUPALS), Higashijima, Akiha Ku, Niigata, Japan; 2 Department of Cardiovascular Sciences, Houston Methodist Research Institute, Houston, Texas, United States of America; 3 J.K.K. Nattraja College of Pharmacy, Komarapalayam, Tamil Nadu, India; 4 Department of Gastroenterology and Hepatology, Niigata University Graduate School of Medical and Dental Sciences, Niigata, Japan; 5 Section of Biochemistry and Cell Biology, Hong Kong University of Science and Technology, Clear Water Bay, Hong Kong SAR, China; 6 Basic studies on second generation functional foods, NUPALS, NUPALS Liaison R/D promotion devision, Higashi-jima 265-1, Akiha-ku, Niigata, Japan, and Changchun University of Chinese Medicine, Bosuo Road #1035 Jingyue Economic Development District, Changchun, RP China; Georgia Regents University, UNITED STATES

## Abstract

Doxorubicin (Dox) is a highly effective antineoplastic drug. However, Dox-induced apoptosis in cardiomyocytes leads to irreversible degenerative cardiomyopathy, which limits Dox clinical application. Schisandrin B (Sch B), a dibenzocyclooctadiene derivative isolated from the fruit of *Schisandra chinensis*, has been shown to protect against oxidative damage in liver, heart and brain tissues in rodents. In current study, we investigated possible protective effects of Sch B against Dox-induced cardiomyopathy in mice. Mice received a single injection of Dox (20 mg/kg IP). Five days after Dox administration, left ventricular (LV) performance was significantly depressed and was improved by Sch B treatment. Sch B prevented the Dox-induced increase in lipid peroxidation, nitrotyrosine formation, and metalloproteinase activation in the heart. In addition, the increased expression of phospho-p38 MAPK and phospho-MAPK activated mitogen kinase 2 levels by Dox were significantly suppressed by Sch B treatment. Sch B also attenuated Dox-induced higher expression of LV proinflammatory cytokines, cardiomyocyte DNA damage, myocardial apoptosis, caspase-3 positive cells and phopho-p53 levels in mice. Moreover, LV expression of NADPH oxidase subunits and reactive oxygen species were significantly less in Sch B treatment mice after Dox injection. These findings suggest that Sch B attenuates Dox-induced cardiotoxicity via antioxidative and anti-inflammatory effects.

## Introduction

Doxorubicin (Dox) is one of the most frequently used anticancer agents in the treatment of both solid and hematologic malignancies. Its therapeutic use is limited by cardiotoxicity categorized as acute or chronic events. The Dox-induced cardiotoxicity is characterized by left ventricular (LV) dysfunction, often leading to congestive heart failure with poor prognosis [1]. The precise cellular mechanisms responsible for this chronic cardiotoxicity of Dox remain enigmatic, but the antitumor activity of Dox is likely to be distinct from the mechanism of its cardiotoxicity [2].

The mechanisms of Dox-induced cardiomyopathy are not fully understood, but a solid body of evidence indicates that oxidative stress and cardiac inflammation are involved [3]. We and others showed previously that both attenuated cardiac cytokine activation and lipid peroxidation might contribute to improved LV function in a mouse model of Dox-induced cardiotoxicity [3, 4]. Dox-induced cardiomyopathy has been linked to apoptosis and DNA damage, free-radical formation, and alterations of calcium metabolism [5]. Accumulating evidence indicates that Dox-induced cardiomyopathy is mainly caused by increased oxidant production, which eventually leads to the apoptotic loss of cardiomyocytes [6–8]. Nevertheless, to date, no single chemical has proven to be able to reduce the deleterious action of Dox. Therefore, the search for an effective and safe antagonist of Dox cardiac toxicity remains a critical issue in both cardiology and oncology.

**Fig 1:**
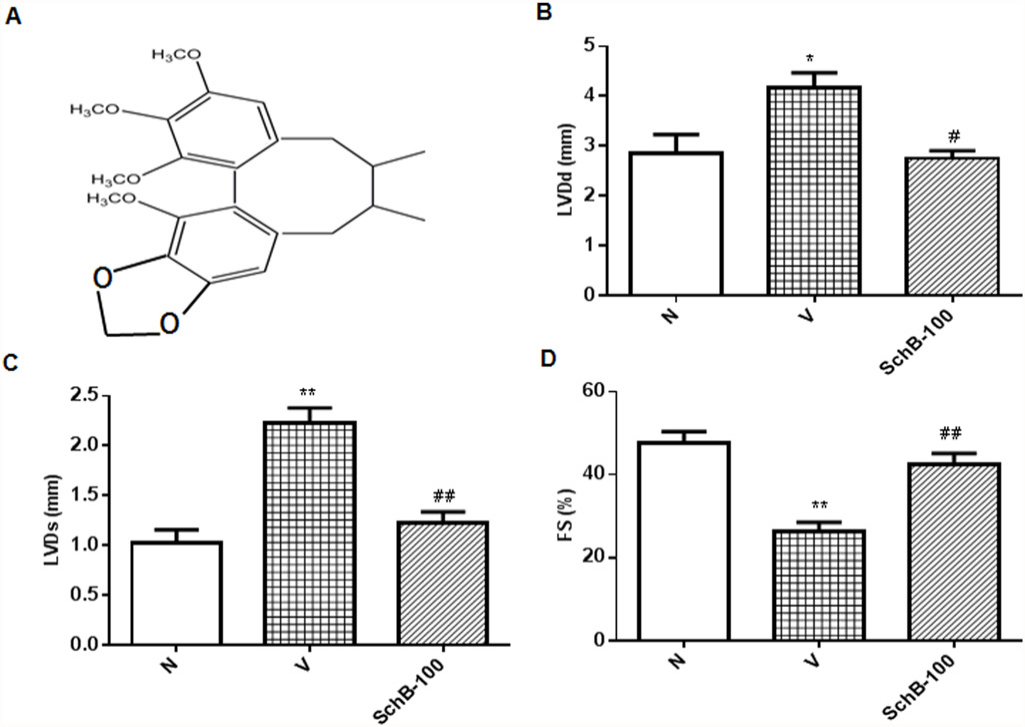
Sch B suppressed doxorubicin-induced cardiac functional loss. (A) Chemical structure of Sch B. (B–D) Mice were treated with a single dose of doxorubicin (20 mg/kg, i.p.) with or without pretreatment of Sch B, followed by analysis of cardiac function of the left ventricle by echocardiography as described in Materials and Methods. Each bar represents means ± S.E. (n = 3–5). **P < 0.01 vs. group N mice; ^#^ *P <* 0.05 and ^##^ *P <* 0.01 vs. group V mice.

In the present study, we focused on the cardioprotective effect of schisandrin B (Sch B), the most abundant dibenzocyclooctadiene lignan (the chemical structure of Sch B as shown in Fig. 1A), isolated from fructus *Schisandra chinensis* (FS), which exert high antioxidant potential both *in vitro* and *in vivo* [9, 10]. FS is a traditional Chinese herb commonly used to treat cough, mouth dryness, spontaneous sweating, dysentery and insomnia. It is clinically used for the treatment of hepatitis and myocardial disorders [11]. It is also a major component herb of many traditional herbal medicines including Shengmai San (SMS). We previously showed SMS effectively prevents cerebral oxidative damage in rat and also in the neuronal cell model [12, 13]. Cardiac and neuroprotective function of FS and the lignans of FS have also been published [9, 10, 14]. Recently, we identified the neuroprotective property of Sch B in scopolamine induced dementia. Previous studies have demonstrated that Sch B protected against myocardial ischemia/reperfusion (I/R) injury and also possesses strong anti-inflammatory property in in-vivo and in-vitro model [9, 15]. In addition, we have showed that Sch B specifically inhibits phosphorylation of p53 through ATR inhibition in *in-vitro* model [16]. Therefore, Sch B is an attractive target ingredient to study the mechanism of cardioprotection by these heart targeting herb or prescriptions.

Hence the present study was undertaken to assess the preventive effects of Sch B on Dox-induced cardiomyopathy. The results of the present study could clarify the role of this herbal drug in the prevention of Dox-induced cardio toxicity, and may shed light on a possible solution to this very serious cardiac complication of Dox.

## Materials and Methods

### Animals

Male C57BL/6 JAX mice (10–12 weeks) were obtained from Charles River Japan Inc., Kanagawa, Japan. Mice were maintained with free access to water and chow throughout the period of study, and animals were treated in accordance with the *Guidelines for Animal Experimentation* of our institute. All animal protocols used in this study were approved by the Institutional Review Board at Niigata University of Pharmacy and Applied Life Sciences.

### Animal protocol

Sch B was kindly provided by Professor Ko at Hongkong University of Science and Technology, and that was isolated from the petroleum ether extract of FS as described by Ip et al [17]. The purity was greater than 95%, as assessed by HPLC. The well-established protocol of Meyers et al [18] was used to produce subacute Dox injury in mice. Dox-induced cardiac dysfunction was induced by injection with Dox (20 mg/kg, i.p.) (Kyowa Hakko Co. Ltd., Tokyo) [3]. After Dox injection, the mice were divided into four groups and received oral administration of Sch B (25 mg/kg/daily; Group- Sch B-25, 50 mg/kg/daily; Group- Sch B-50 and 100 mg/kg/daily; Group-Sch B-100) and Vehicle (Group-V) for 5 days. Age matched C57BL/6 JAX mice injected with saline was used as normal control (group-N).

### Transthoracic echocardiography

Two-dimensional echocardiography studies were performed in anesthetized mice (pentobarbital, 50 mg/kg, i.p) to evaluate cardiac function using an echocardiographic machine equipped with 7.5 and 12 MHz transducers linked to an ultrasound system (SSD-5500; Aloka, Tokyo, Japan) by an experienced echocardiographic analyst who did not have knowledge of mouse genotype or previous treatment. The short-axis view of the left ventricle was recorded to measure the LV dimension in systole (LVDs) and diastole (LVDd) as well as the percent fractional shortening (% FS) and percent ejection fraction (% EF). Animals were sacrificed by cervical dislocation and all efforts were made to minimize suffering. Hearts were harvested for analysis from control and Dox treated mice. The left ventricle was quickly dissected and cut into two parts. One part was immediately transferred into liquid nitrogen and then stored at -80°C for protein analysis. The other part was either stored in 10% formalin or at -80°C after the addition of Tissue-Tek OCT compound (Sakura Co. Ltd., Tokyo, Japan) for histopathological and immunohistochemical analysis.

### Histopathological studies

The LV portion of heart tissues was fixed in 10% neutral buffered formalin. Sections of 3–5 μm thickness were stained with haematoxylin and eosin (HE) for histological examination. A histomorphological evaluation of all the heart sections was carried out in a blinded fashion by a pathologist who was unaware of the treatment groups.

### Single cell gel electrophoresis assay

DNA damage was detected and quantified using the alkaline single cell gel electrophoresis assay comet assay [3, 19]. Cardiomyocytes isolated as described previously [20] were mixed with 0.8% low melting agarose at 38°C and spread on fully frosted slide. After solidification, slides were immersed in lysis buffer (2.5 M NaCl, 100 mM Na_2_-EDTA, 1%Triton X-100,and 10% DMSO) for 1 hour at 4°C and electrophoresed in alkaline buffer (300 mM NaOH, 1 mM Na_2_-EDTA, pH 13) using 25 V, 400 mA for 30 minutes. After electrophoresis the slides were stained with SYBR-Green-II dye and observed under a fluorescence microscope at 200x magnification. At least images of 50 cells per slide were captured (CIA-102, Olympus, Tokyo, Japan). The digital imaging casp-software (http://casp.sourceforge.net/) was used to measure the indexes of DNA damage. Tail length and tail moment was selected as the parameter to quantify the extent of DNA damage. [19]

### Terminal deoxynucleotidyl transferase-meditated dUTP nick-end labeling (TUNEL) assay

Frozen LV tissues embedded in OCT compound were cut into 4-μm thick sections and fixed in 4% paraformaldehyde (pH 7.4) at room temperature. TUNEL assay was performed as specified in the *in situ* apoptosis detection kit (Takara Bio Inc, Shiga, Japan) and sections were examined under fluorescence microscopy at 200x magnification (CIA-102, Olympus, Tokyo, Japan). For each animal, five sections were scored for apoptotic nuclei. For each slide 10 fields were randomly chosen, and by using a defined rectangular field area, a total of 100 cells per field were counted. The percentage of total myocytes that were TUNEL positive (apoptotic index) was then calculated. This evaluation was performed by one person who was blinded to treatment group.

### Immunofluorescence

For immunofluorescence assay, tissues were fixed in 10% buffered formaldehyde solution and embedded in paraffin. Sections were underwent microwave antigen retrieval, blocked with 10% goat serum in phosphate buffer saline, and incubated with polyclonal rabbit anti-caspase-3 antibody (Cell Signaling Technology). The primary antibody bound sites were visualized with fluorescein isothiocyanate conjugated secondary antibody (Sigma Aldrich) for a fluorescence microscopic observation at 400x magnification (CIA-102, Olympus, Tokyo, Japan) [3, 21].

### *In situ* detection of superoxide production in hearts

To evaluate *in situ* the superoxide production from hearts, unfixed frozen cross sections of the specimens were stained with dihydroethidium (DHE; Molecular Probe, OR) according to the previously validated method [3, 22–24]. In the presence of superoxide, DHE is converted to the fluorescent molecule ethidium, which intercalates with DNA to visualize the nuclei. Briefly, the unfixed frozen tissues were cut into 10-μm-thick sections and incubated with 10 μM DHE at 37°C for 30 min in a light-protected humidified chamber. Fluorescence images were obtained using a fluorescence microscope equipped with a rhodamine filter.

### Protein analysis

Protein lysate was prepared from heart tissue as described previously [25]. The total protein concentration in samples was measured by the bicinchoninic acid method. For western blotting experiments, 30 μg of total protein was loaded and proteins were separated by SDS-PAGE (200 V for 40 min) and electrophoretically transferred to a nitrocellulose filters (semi-dry transfer at 10 V for 30 min). Filters were then blocked with 5% non-fat dry milk in Tris buffered saline (20 mM Tris, pH 7.6, 137 mM NaCl) with 0.1% Tween 20, washed, and then incubated with primary antibody. Primary antibody included: rabbit polyclonal phosho-p38 MAPK, phosphor-mitogen activated protein kinase-activated protein kinase 2 (MAPKAPK-2), a downstream effector of p38 MAPK and phopho-p53 (Cell Signaling Technology Inc., MA, USA), rabbit polyclonal p47phox and p67phox, goat polyclonal gp91pox and glyceraldehyde-3-phosphate dehydrogenase (GAPDH) (Santa Cruz Biotechnology Inc., CA, USA). After incubation with the primary antibody, the bound antibody was visualized with horseradish peroxidase-coupled secondary antibodies (Santa Cruz Biotechnology) and chemiluminescence developing agents (Amersham Biosciences, Buckinghamshire, UK) and detected by X-ray film exposure. The level of expression of each protein in control WT mice was taken as one arbitrary unit (AU). For western blotting analysis, all primary and secondary antibodies were used at a dilution of 1:1000 and 1:5000, respectively. Films were scanned after development and fixation treatments and the expression levels of each protein were quantified by densitometric analysis of corresponding band using Scion image software (Epson GT-X700; Tokyo, Japan).

### Cytochrome c reduction assay

NADPH dependent superoxide production was examined using superoxide dismutase (SOD)-inhibitable cytochrome *c* reduction [21]. Total protein from myocardial tissue (final concentration 1 mg/ml) was distributed in 96-well plates (final volume 200 μl/well). Cytochrome *c* (500 μmol/l) and NADPH (100 μmol/l) were added in the presence or absence of SOD (200 U/ml) and incubated at room temperature for 30 min. Cytochrome *c* reduction was determined by the absorbance at 550-nm wavelength using a microplate reader. Superoxide production was calculated from the difference between the absorbances with and without SOD using the extinction coefficient of ferrocytochrome c, i.e., 21.0 mM L^−1^ cm^−1^.

### Antioxidant assay

The heart tissue was homogenized (10% w/v) in ice-cold sodium phosphate buffer (30 mM, pH 7.0) at 9500 rpm x 3 times with intervals of a few sec between the runs and the supernatant fraction was used for a different antioxidant enzyme activity assay. The quantitative measurement of malondialdehyde (MDA), end product of lipid peroxidation, in heart homogenate was performed according to the method of Ohkawa et al. [34]. Reduced glutathione (GSH) was determined by the Ellman method [35], which is based on the development of a yellow color due to the reaction of DTNB with compounds containing sulfhydryl groups.

### Electron spin resonance (ESR) spectroscopy

The formation of hydroxyl radical (·OH) was detected with 5,5′-dimethyl-1-pyrroline-1-oxide (DMPO) as a spin trap [3, 21]. The hearts were homogenized in cold PBS (100 mg hearts/ml), incubated with 200 μmol/L Dox (group V and Sch B treated) or without Dox (group N) for 10 minutes, and then added to 0.05 ml of 9.0 mol/L DMPO. The generation of hydroxyl radicals was detected as DMPO-•OH adduct using JEOL JES-TE 200 ESR spectrometer. The DMPO-OH signal intensity was normalized for quantification using Mn^2+^ signal as the reference. The spectrophotometer settings were as follows: microwave frequency, 9.43 GHz; microwave power, 8.0 mW; modulation amplitude, 0.1 mT; time constant, 0.03 seconds; sweep time, 30 seconds and centre fields, 342/332 mT.

### Estimation of nitrite

The accumulation of nitrite, an indicator of the production of nitric oxide, was determined using Griess reagent as described by Green et al. [26]. The heart tissue homogenate supernatant was added with equal volume of Griess reagent and incubated for 10 min at room temperature in the dark and absorbance was determined at 540 nm (Shimazu model UV/Vis spectrophotometer). The concentration of nitrite in the supernatant was determined from the standard curve prepared for sodium nitrite and expressed as μM/mg protein.

### RNA Extraction

Heart tissues were preserved by immersion in RNAlater (Ambion Inc., Austin, TX) immediately after sampling. The extraction of total RNA was performed after homogenization by using Ultra TurraxT8 (IKA Labortechinik, Staufen, Germany) in TRIzol reagent (invitrogen Corp., CA) according to the standard protocol. Synthesis of cDNA was performed by reverse transcription using total RNA (2 μg) as a template (Super Script II; Invitrogen Corporation, Carlsbad, CA).

### Gene expression analysis by real time RT-PCR

Gene expression analysis was performed by real time reverse transcription polymerase chain reaction (RT-PCR) (Smart cycler; Cepheid, Sunnyvale, CA) using cDNA synthesized from the heart samples. Primer sequences were as follows: Interleukin (IL)-1β, IL-6, tumor necrosis factor (TNF)–α, Matrix metallopeptidase (MMP) 2 and 9, and GAPDH. Real time RT-PCR by monitoring with TaqMan probe (TaqMan Gene expression assays; Applied Biosystems, Foster City, CA) was performed according to the following protocol: 600 seconds at 95°C, followed by thermal cycles of 15 seconds at 95°C, and 60 seconds at 60°C for extension. Relative standard curves representing several 10 fold dilutions (1:10:100:1000:10,000:100,000) of cDNA from heart tissue samples were used for linear regression analysis of other samples. Results were normalized to GAPDH mRNA as an internal control and are thus shown as relative mRNA levels.

### Statistical analysis

Data are represented as means ± standard error (S.E.). Statistical analysis amongst groups was determined by *t*-test or by one-way analysis of variance followed by Tukey’s method. Differences were considered as statistically significant at *P* < 0.05.

## Results

### Sch B attenuated ventricular and morphological remodeling

**Fig 2:**
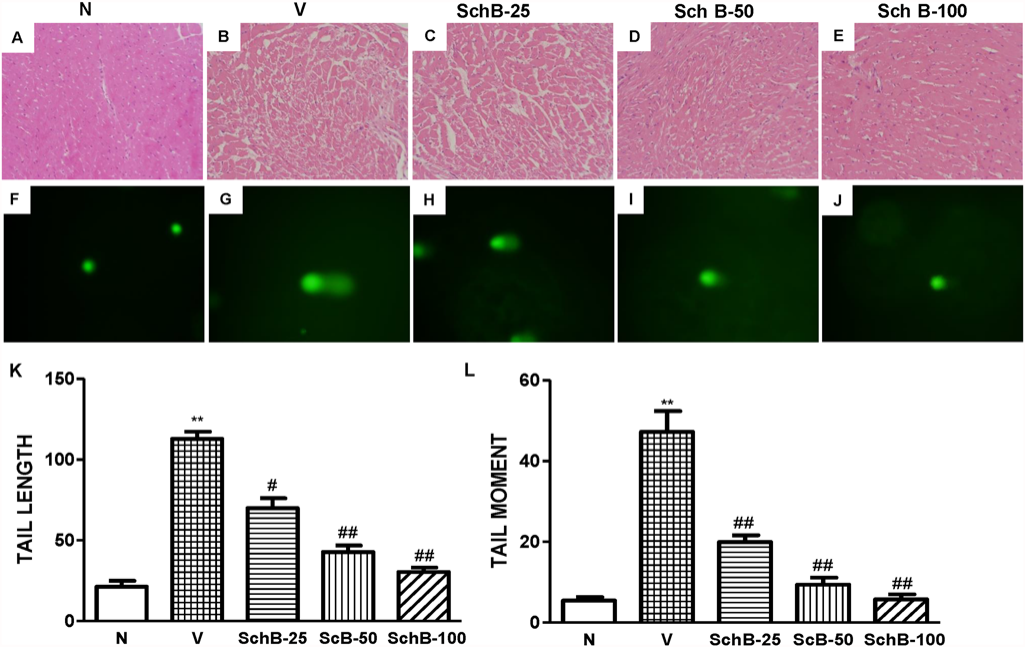
Sch B suppressed the cardiac morphological changes and DNA damage. Typical photomicrographs of heart tissue from group N (A), group V (B) and group Sch B (C–E). Cytoplasmic vacuolation and myofibrillar disorganization in the doxorubicin group were effectively attenuated Sch B treatment (haematoxylin and eosin ×200). (F–J) Representative photomicrographs of DNA damage 5 days after Dox injection. DNA damage detected by single cell gel electrophoresis assay (Comet assay) in cardiomyoctes of all groups at 200x magnification. (K–L) Quantification of cardiomyocyte DNA damage analyzed by digital imaging casp-software (http://casp.sourceforge.net/). **P < 0.01 vs. group N mice; ^#^ *P <* 0.05 and ^##^ *P <* 0.01 vs. group V mice.

Echocardiographic studies in group V mice showed increased LVDd and LVDs, and reduced FS indicating impaired systolic function compared with that in group N mice (Fig. 1B-D). Treatment with Sch B significantly decreased the LVDd and increased FS compared with those in group V mice (Fig. 1B-D). Dox-induced morphological changes in cardiac tissues were observed by light microscopy, as shown in Fig. 2A-E. There were significant morphological changes occurred in group V mice (Fig. 2B) after Dox treatment when compared to the normal morphology shown in Fig. 2A. Cross-sections of cardiac tissue of the mice from group V showed cytoplasmic vacuolization and myofibrillar disorganization. These changes observed in the myocardium of group V mice were successfully prevented in Sch B treated group and nearly recovered to normal (Fig. 2B-E).

### Sch B inhibits DNA damage, TUNEL and caspase-3 activation

**Fig 3:**
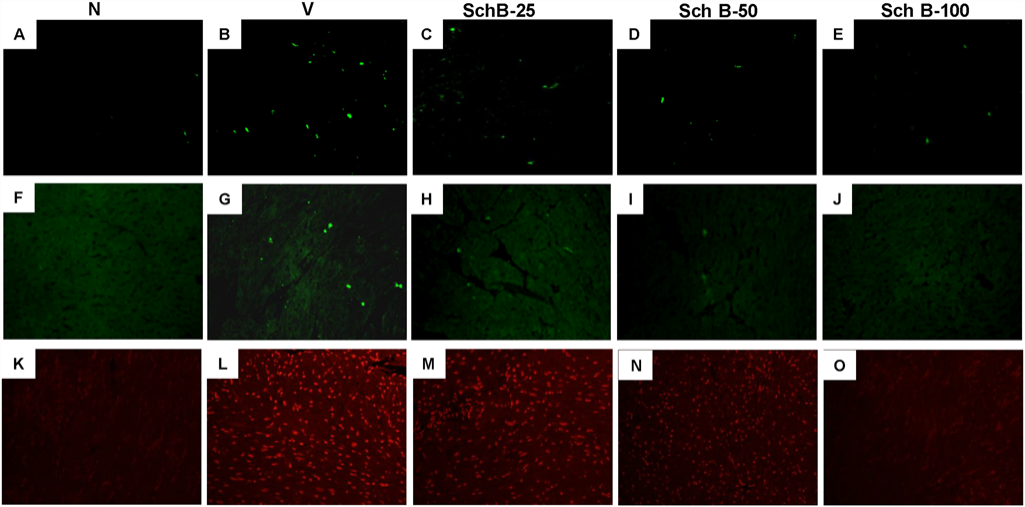
Sch B inhibits cardiac apoptosis, caspase-3 positive cells and superoxide production. (A–E) Myocardial tissue sections TUNEL stained for apoptotic nuclei in group N (A), group V (B) and Sch B treated mice (C–E) at 200x magnification. (F–J) Representative photomicrographs of LV sections showing caspase-3 immunofluoroscence identified by immunohistochemical staining with anti-capsase-3 antibody, group N (F), group V (G) and Sch B treated mice (H–J) (400x); Intense immunofluoroscence was detected in LV sections of group V mice. (K–O) In situ superoxide production (bright area) using dihydroethidium (DHE) staining in hearts of group N (K), group V (L) and Sch B treated mice (M–O) at 200x magnification.

DNA damages were detected and quantified using the alkaline single cell gel electrophoresis assay [3, 19]. The fragments of DNA produced by DNA strand break forms comet tail under the electrophoresis condition and thus both the length and the fractional area of the comet are proportional to DNA damages. The DNA damage measured in terms of tail moment and tail length was significantly increased in group V mice compared to group N mice (Fig. 2F, G, K and L). In contrast, treatment with Sch B significantly inhibited the DNA damages compared with group V in dose dependent manner (Fig. 2G, H, I, J, K and L). Expectedly, the number of TUNEL-positive cells and caspase-3 positive cells were markedly increased in LV sections of group V compared to group N (Fig. 3A, B, F and G). Interestingly, Dox-induced TUNEL positive cells and caspase-3 positive cells were attenuated by Sch B treatment in dose dependent manner (Fig. 3A-J).

### Sch B attenuates myocardial expression of phospho-p53 and 3-NT

**Fig 4:**
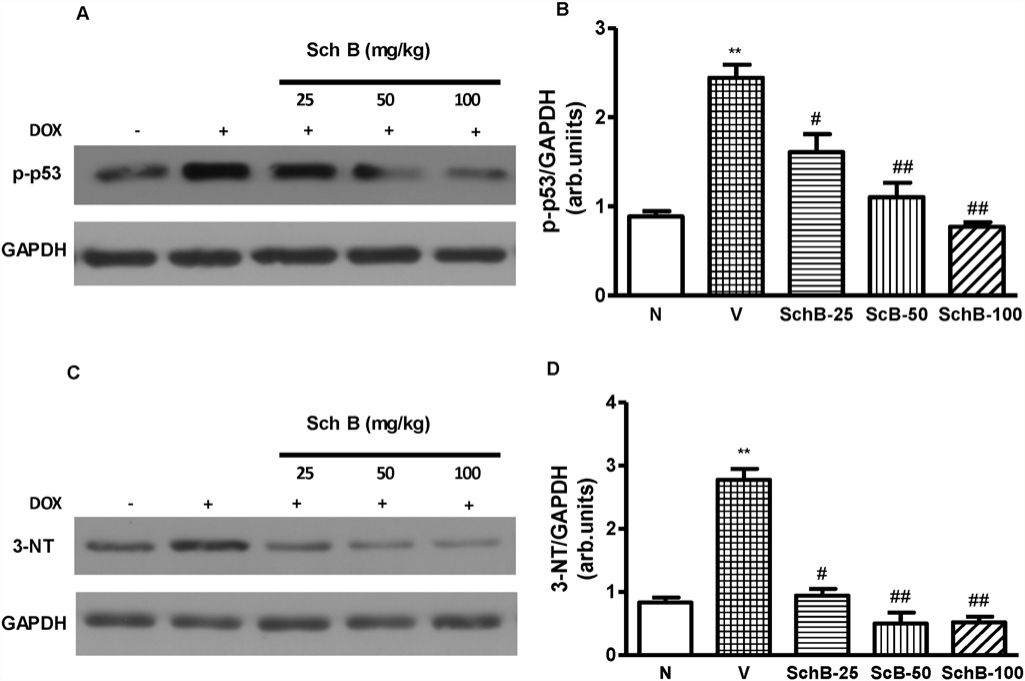
Sch B attenuated LV p-53 and 3-NT expression. (A–D) Representative Western immunoblots and densitometry analysis using Scion image software for p-p53 (A, B), and 3-NT (C and D), normalized against GAPDH. Each bar represents means ± S.E. (n = 4–5). **P < 0.01 vs. group N mice; ^#^ *P <* 0.05 and ^##^ *P <* 0.01 vs. group V mice.

Immunoblot analysis revealed that LV expression levels of phospho-p53 and 3-NT were significantly increased in group V compared to group N mice (Fig. 4A-D). However, Sch B treatment dose dependently attenuated the phosphorylation levels of p53 and 3-NT when compared to group V (Fig. 4A-D).

### Sch B attenuated Dox-induced myocardial expression of phospho-p38 MAPK and phospho-MAPKAPK-2

**Fig 5:**
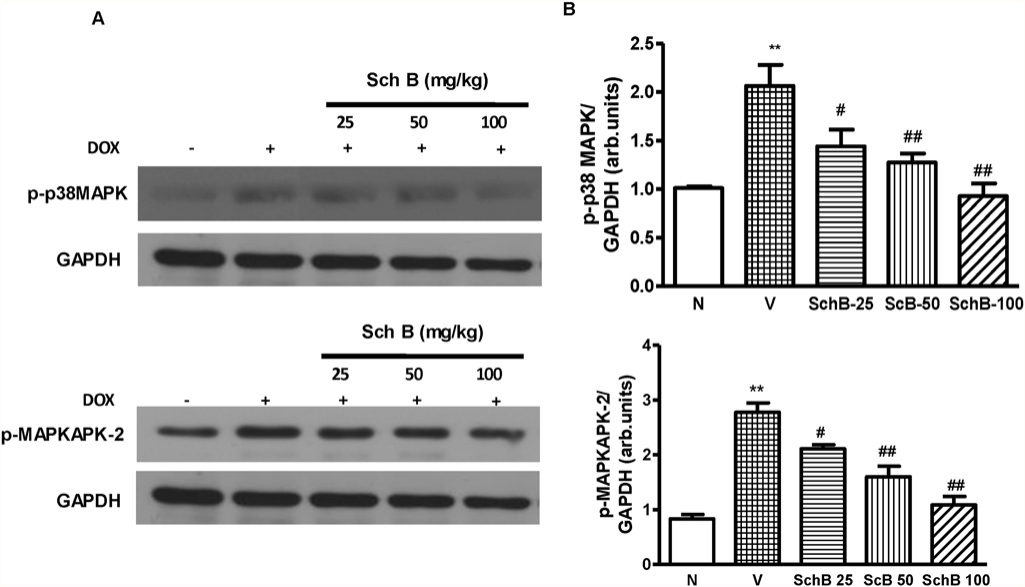
Reduced p38 MAPK activity in Sch B treated mice at 5 days after Dox injection. (A–D) Representative Western immunoblots and densitometry analysis using Scion image software for p-p38 MAPK (A and B) and p-MAPKAPK-2 (C and D). Blots were normalized against GAPDH. Each bar represents means ± S.E. (n = 3–5). **P < 0.01 vs. group N mice; ^#^ *P <* 0.05 and ^##^ *P <* 0.01 vs. group V mice.

Immunoblot analysis revealed that LV expression of both phospho-p38 MAPK and phospho-MAPKAPK-2, a well described substrate of p38 MAPK, were significantly increased in group V compared to group N (Fig. 5A-D). However, treatment with Sch B significantly attenuated the Dox-induced p38 MAPK and MAPKAPK-2 activation in dose dependent manner (Fig. 5A-D).

### Sch B inhibits hydroxyl radical production detected by ESR

**Fig 6:**
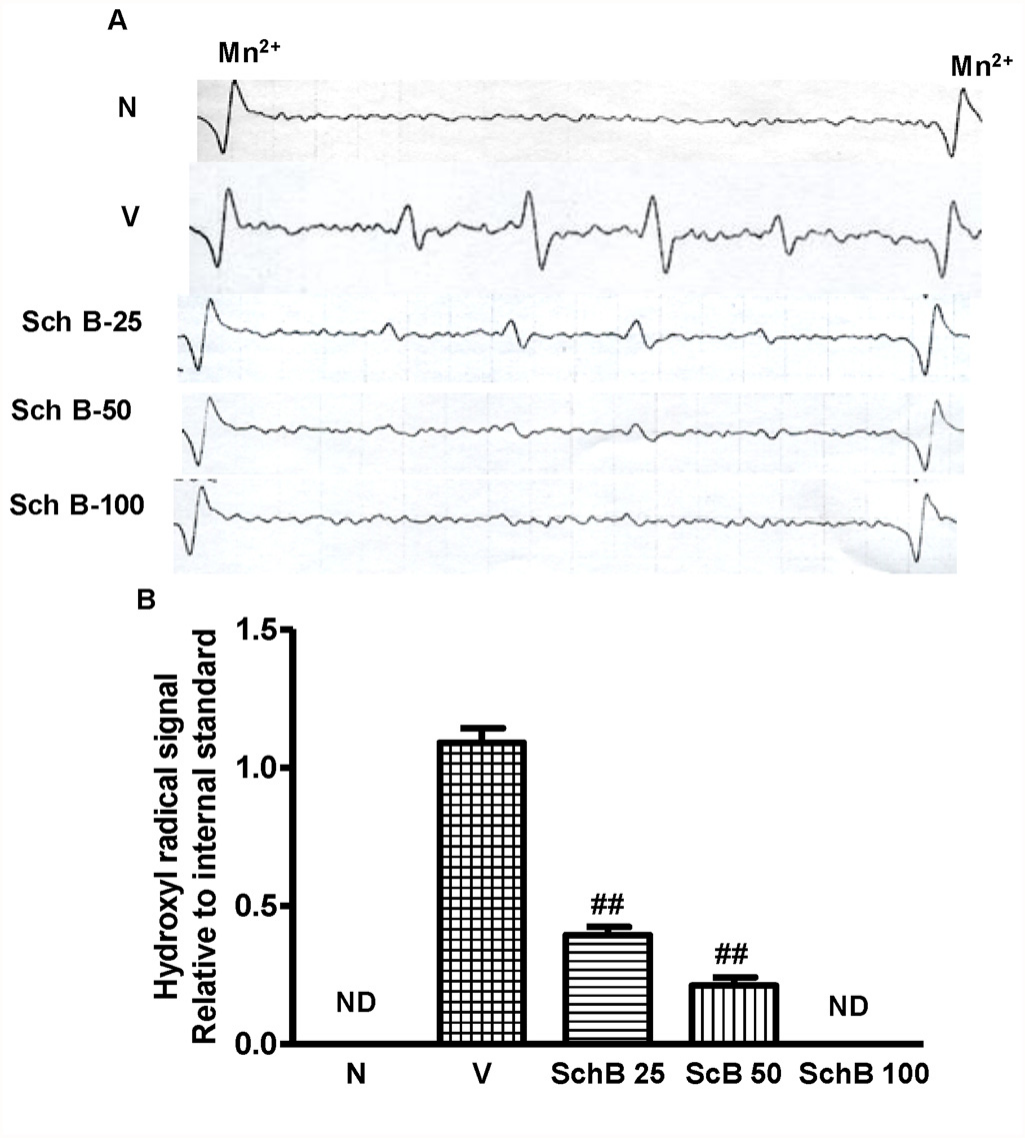
Sch B attenuates Dox-induced hydroxyl radical. (A, B) Representative ESR spectra and analysis of the hydroxyl radical signal relative to the internal standard of manganese ion. Hydroxyl radical signals were not detected (ND) in hearts of group N and group Sch B-100. Mn (3) and Mn (4) indicate the internal standard signals of manganese ion (Mn^2+^).

DMPO spin trapping analyses showed the formation of hydroxyl radical signals in the group V heart homogenates and the signals were significantly small in the Sch B treated heart homogenates (Fig. 6A and B). No signals were detected in the heart homogenates of group N mice.

### Sch B modulates glutathione level and attenuates MDA and nitrite level

**Fig 7:**
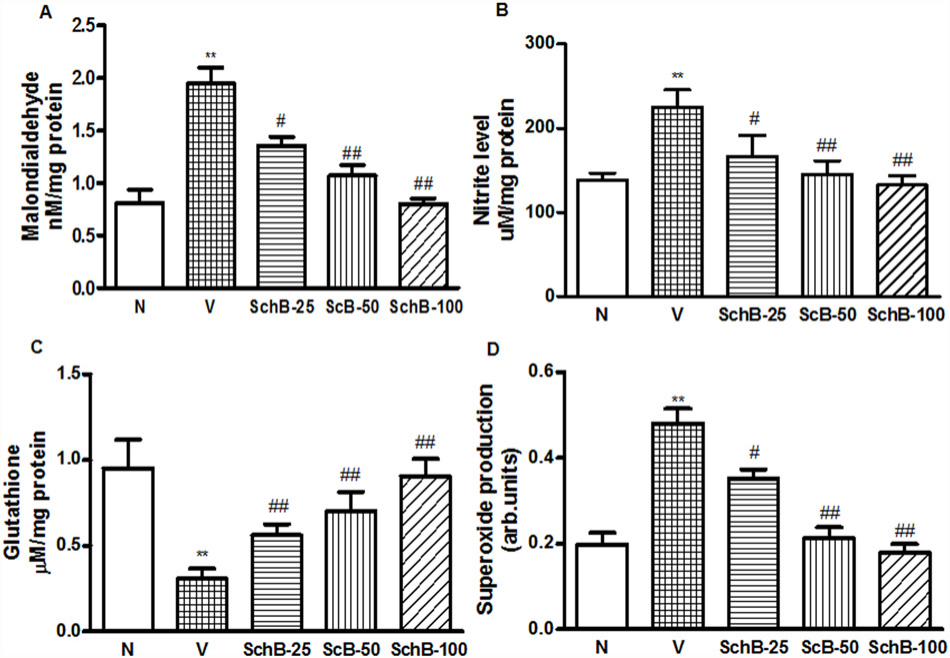
Sch B attenuates Dox-induced myocardial ROS and oxidative stress, and enhance antioxidant enzyme. (A) MDA level, (B) Nitrite level, (C) Glutathione level and (D) Superoxide production by LV homogenates. **P < 0.01 vs. group N mice; ^#^ *P <* 0.05 and ^##^ *P <* 0.01 vs. group V mice.

The treatment of mice with Dox resulted in a significant increase in MDA and nitrite levels, (Fig. 7A and B) and significant decreases in GSH levels in LV (Fig. 7C). The treatment of Dox mice with Sch B recovered the GSH level in dose dependent manner. Sch B also significantly decreased the MDA and nitrite levels compared with those of the Dox-treated group, as shown in Fig. 7A-C.

### Sch B attenuates Dox induced ROS and oxidative stress

**Fig 8:**
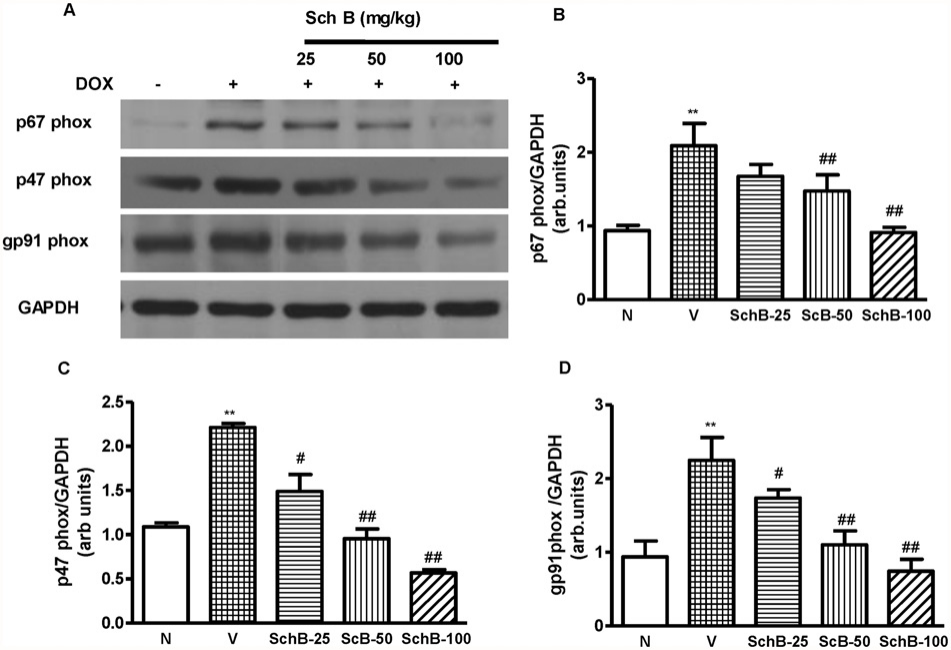
Sch B inhibits Dox induced NADPH oxidase subunits. (A–D) Representative Western immunoblots and densitometry analysis using Scion image software for p47phox, p67phox and gp91phox in group N, group V and Sch B treated mice; Blots were normalized against GAPDH. Each bar represents means ± S.E. (n = 3–5). **P < 0.01 vs. group N mice; ^#^ *P <* 0.05 and ^##^ *P <* 0.01 vs. group V mice.

DOX cardiomyopathy has been reported to be associated with enhanced ROS generation and oxidative stress [27]. Intracellular superoxide radical level was determined by DHE staining as in reported elsewhere [22–24]. Fig. 3L shows the increased intracellular red fluorescence due to the intercalation of ethidium into DNA in the heart of group V mice compared to group N mice. The Dox-induced enhancement in ethidium fluorescence was dose dependently inhibited by Sch B treatment (Fig. 3M-O), indicating the superoxide radical production was inhibited by Sch B to reduce overall oxidative stress. Indeed, NADPH-dependent O_2_^-^ production in LV homogenates was significantly increased in the hearts of group V mice compared to group N mice (Fig. 7D) but the ROS production was significantly lower in the hearts of Sch B treated group compared to group V (Fig. 7D). Because NADPH oxidase is the main source of ROS in the cardiovascular tissues [28], we next measured the protein expression of p47phox, p67phox, and gp91phox in the hearts of group N, V and Sch B treated animals. Myocardial expression of p47phox, p67phox and gp91phox were significantly elevated in group V mice compared to group N (Fig. 8A and B). However, Sch B treatment attenuated the elevated NADPH oxidase subunits in dose dependent manner. These data suggest that the NADPH oxidase mediated superoxide radical production may be the primary factor of oxidative stress, which caused the cardiac damage in Dox mice.

### Sch B attenuates cytokine production, and expression of MMP-2, and 9

**Fig 9:**
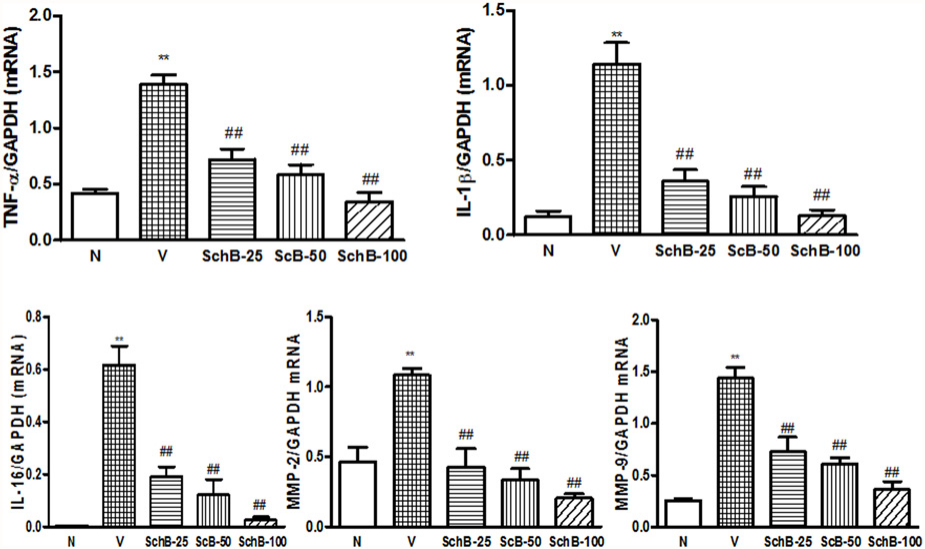
Sch B inhibits the Dox-induced messenger RNA expression of LV cytokines and MMP levels 5 days after Dox injection. (A–E) mRNA expression of TNF-α (A), IL-1β (B), IL-16 (C), MMP-2 (D) and MMP-9 (E) in group N, group V and Sch B treated mice. The expression level of each sample was expressed relative to the expression level of GAPDH gene. Each bar represents means ± S.E. (n = 3–5). **P < 0.01 vs. group N mice; ^#^ *P <* 0.05 and ^##^ *P <* 0.01 vs. group V mice.

We examined proinflammatory cytokine production in myocardial tissue after Dox injection (Fig. 9A-E). The mRNA expression of TNF-α, IL-1β, IL-6, MMP-2 and 9 were markedly elevated in group V compared to group N. However, after treatment with Sch B, these cytokines and MMP levels in the myocardium were significantly attenuated in dose dependent manner (Fig. 9A-E).

## Discussion

The mechanism of Dox-induced cardiomyopathy has been studied rigorously since this complication was first reported but the solution to this potentially fatal complication has not been determined [29, 30]. Here, we showed that treatment with the Sch B leads to cardioprotection against Dox in mice. We showed that Sch B inhibited the Dox induced p53 phosphorylation, proinflammatory cytokines, ROS production and oxidative stress, nitrative stress, cardiomyocyte DNA damage, TUNEL-positive cells in the myocardium, and caspase-3 positive cells, which were associated with cardiac dysfunction. These results provide adequate evidence that Sch B possesses beneficial properties against Dox toxicity, and raises this herbal agent a potential therapeutic adjuvant that may prevent the heart from the serious side effect of Dox.

Dox is one of the most important anticancer agents. However, clinical use of Dox is limited because of its cardiotoxicity. For example, Olson et al [31] have shown that mice treated with 20 mg/kg Dox developed cardiac failure. Although the precise mechanisms whereby Dox induces myocardial injury have not been fully documented, it is widely accepted that the cardiac toxicity of Dox is mediated by ROS [32, 33]. FS is clinically used for the treatment of hepatitis and myocardial disorders and possesses strong antioxidant activities [11]. It is also a major component herb of many traditional herbal medicines including SMS. Cardiac and neuroprotective function of FS and the lignans of FS have also been published [9, 10, 14]. Previous studies have demonstrated that Sch B a major lignan of FS protected against myocardial ischemia/reperfusion (I/R) injury and also possesses strong anti-inflammatory property in *in-vivo* and *in-vitro* model [9, 15]. However the protective function of Sch B in Dox induced cardiac injury is remained unclear. On the basis of these studies, we focused our attention on the protective mechanisms of Sch B in cardiac dysfunction, by observing the cellular signals including cytokine production, ROS production, oxidative/nitrative stress, DNA damage and apoptosis in the mice injected with a single dose of Dox (20 mg/kg) in this study.

Administration of a single dose of Dox to mice has been validated extensively in the study of Dox-induced cardiac dysfunction in vivo [34, 35]. In agreement with others and our previous studies, Dox treated mice were found to display severe systolic and diastolic LV dysfunction, resulting in impaired cardiac dysfunction [3, 35]. In Sch B treated mice, however, the LV function was markedly improved relative to the Dox treated mice as were shown in the results of enhanced systolic and diastolic performance measured by echocardiography (Fig. 1B-D). Thus, we conclude that Sch B plays a pivotal role in LV dysfunction due to Dox-induced cardiomyopathy. To further analyze the mechanisms involved, we characterized cardiac oxidative/nitrative stress, inflammatory response, DNA damage and apoptosis, which are all known to be relevant in this disease.

p38 MAPK family, is activated by physical and chemical stress factors, playing roles in many physiological reactions such as growth promotion, apoptosis, oxidative stress, and vasoconstriction [36–39]. Previous study has demonstrated that Dox activates p38 MAPK in cultured cardiomyocytes [40] and recently, it has also been shown elsewhere Sch B inhibits the phosphorylation of p38 MAPK and pro-inflammatory cytokines in dose dependent manner [15]. Consistent with these findings, we have found that both p38 MAPK and its downstream effector MAPKAPK-2 were activated in hearts of mice after Dox injection, and the activation was suppressed by Sch B in dose dependent manner. Dox-induced cardiomyopathy is also associated with increased levels of the MMP2- and 9, and pro-inflmammatory cytokines TNF-α, IL-1β, and IL-6 as similar to the results observed in kidney [35]. This cardiac inflammation is linked with decreased LV function, not only in the Dox-induced cardiomyopathy, but also in diabetic cardiomyopathy, pressure overload, and dilated cardiomyopathy [41, 42]. The p38 MAPK is a key regulatory pathways for many genes, including those regulating TNF-α, IL-1β and TGF-β [43]. In our study, cytokine production and MMP levels were increased in mice 5 days after Dox injection, and these Dox-induced increases in cytokine production and MMP levels were suppressed by Sch B treatment. These results indicate that Sch B inhibits cardiac inflammation and dysfunction after Dox injection, possibly through modulation of MAPK signaling pathways although the precise mechanism remains to be determined.

Oxidative stress and inflammation are well known as the causative factors of DNA damage induction and apoptosis, and is also implicated in Dox-induced cardiomyopathy [5, 27]. Recently, we have reported that p38 MAPK is associated with the onset of apoptosis, DNA damage, ROS production and cardiac remodeling in Dox injected mouse hearts [3]. DNA damage plays an important role in anthracycline-induced lethal cardiac myocyte injury through a p53 pathway [44]. It is reported p38 MAPK phosphorylates p53 in various models [45, 46]. Previous studies have implicated the p53 pathway in Dox-induced cardiotoxicity. For example, reduced levels of cardiomyocyte apoptosis and concomitant improvements in cardiac function were observed in Dox treated p53 null mice compared with their wild-type littermates [47]. Consistent with these studies, we observed massive DNA damage, a marked increase in cardiomyocyte apoptosis and caspase-3 positive cells in mice after Dox injection. The reduction in apoptosis and less DNA damage observed in Sch B treated cardiac tissue may be a consequence of reduced phosphorylation of p53_._ The precise mechanism by which Sch B modulates p53 phosphorylation remains to be determined. Moreover, Masashi et al. have shown that Dox cardiotoxicity is mediated by oxidative DNA damage-ATM-p53-apoptosis pathway *in vitro* and *in vivo*. It is therefore possible that Sch B treatment prevents the oxidative stress induced DNA damage and cardiac apoptosis associating with Dox-induced cardiomyopathy possibly by modulating the p38 MAPK-ATM-p53 axis.

Indeed the property of Sch B found in the present study is the potential antioxidant activity. Kalyanaraman and colleagues have reported that scavenging ROS protects against Dox-induced cardiac apoptosis [48, 49]. Similarly cardiac specific overexpression of antioxidant genes protected mice from Dox-induced cardiac dysfunction [50]. NADPH oxidase is a multicomponent enzyme and the major source of oxidative stress in various diseases. Upon stimulation, the cytosolic complex migrates and assembles with the membrane subunits to form an active oxidase capable of producing superoxide anion [51]. Recently, we have reported that NADPH oxidase is enhanced in Dox heart and it is inhibited by DN p38α MAPK [3]. In addition, we have also found that Sch B treatment improved the antioxidant capacity of scopolamine treated brain tissue in mice. In the present study, we observed that, the myocardial expression of p47phox, p67phox, and gp91phox were significantly suppressed in Sch B treated mice relative to vehicle treated mice at 5 days after Dox injection. Moreover, Dox treated mice had higher level of ROS content after Dox injection relative to Sch B treated mice. In addition, Dox treatment resulted in a significant increase of MDA, an important marker for lipid peroxidation, 3-NT and nitrite levels in the heart and a reduction of GSH activity [52]. Treatment with Sch B not only preserved the reduced GSH level induced by Dox but also elevated to a level comparable to that of normal control mice and inhibited the MDA formation and reduced 3-NT and nitrite level to the normal level. In this regards, it is noteworthy that Sch B may regulate NADPH oxidase activation and thus ROS production. It is yet unclear the enhanced expression of antioxidant enzyme in Dox-induced cardiomyopathy is due to the direct effect of Sch B or indirect through the modulation of cellular redox state. The precise mechanism remains to be determined. In this study, cardiac dysfunction, the expression of proinflammatory cytokines, and the apoptosis that were observed after Dox injection were modulated by the interaction between oxidative stress and the p38 MAPK pathway, and these complications were prevented by Sch B treatment. Therefore, these results provide a new insight into Dox-induced cardiomyopathy in the clinical setting.

Taken together our results suggest that Sch B protects Dox-induced acute toxicity by enhancing cellular antioxidant potential through expression of antioxidant enzymes and inhibition of ROS production, following DNA damage, apoptotic cell death, and cardiac inflammation to prevent Dox-induced cardiomyopathy and ameliorates cardiac dysfunction. Since the oxidative stress is a basic pathology of Dox injury, the natural antioxidant molecules carrying additional functions such as anti-inflammatory properties should be more promising for the treatment of cardiovascular disease. Hence, our present findings suggest that Sch B will provide complementary advantage for the treatment cardiovascular diseases such as Dox cardiomyopathy. Further clinical studies are mandatory to determine whether Sch B is really cardioprotective in the setting of anticancer therapy using Dox or related chemotherapeutic agents.
